# Current Awareness on Comparative and Functional Genomics

**DOI:** 10.1002/1097-0061(20000630)17:2<159::AID-YEA8>3.0.CO;2-7

**Published:** 2000


**Yeast**
*Yeast* 2000; **17:** 159–166

### Current awareness on comparative and functional genomics

### 1 Reviews & symposia

Abu Kwaik Y, Harb OS. 1999. Univ Kentucky, Albert B Chandler Med Ctr, Dept Microbiol & Immunol, Lexington, Ky 40536, USA. Phenotypic modulation by intracellular bacterial pathogens: Review. *Electrophoresis* **20:** (11) 2248.

Andersson JO, Andersson SGE. 1999. Uppsala Univ, Dept Mol Evolut, Biomed Ctr, Box 590, SE-75124 Uppsala, Sweden. Insights into the evolutionary process of genome degradation. *Curr Opin Genet Dev* **9:** (6) 664.

Aravind L, Subramanian G. 1999. Texas A&M Univ, Dept Biol, College Station, Tx 77843, USA. Origin of multicellular eukaryotes: Insights from proteome comparisons. *Curr Opin Genet Dev* **9:** (6) 688.

Baba Y. 1999. Univ Tokushima, Fac Pharmaceut Sci, Dept Med Chem, Tokushima 770 8505, Japan. Capillary affinity gel electrophoresis: New technique for specific recognition of DNA sequence and the mutation detection on DNA (Review). *J Biochem Biophys Methods* **41:** (2-3) 91.

Baker BF, Monia BP*. 1999. *Isis Pharmaceut, Dept Mol Pharmacol, 2292 Faraday Ave, Carlsbad, Ca 92008, USA. Novel mechanisms for antisense-mediated regulation of gene expression (Review). *Biochim Biophys Acta* **1489:** (1) 3.

Bennett CF, Cowsert LM. 1999. Isis Pharmaceut, 2280 Faraday Ave, Carlsbad, Ca 92008, USA. Antisense oligonucleotides as a tool for gene functionalization and target validation (Review). *Biochim Biophys Acta* **1489:** (1) 19.

Borinskaya SA, Yankovsky NK. 1999. Russian Acad Sci, Inst Gen Genet, RU-117333 Moscow, Russia. Structure of prokaryotic genomes. *Mol Biol-Engl Tr* **33:** (6) 831.

Brent R. 2000. Inst Mol Sci, 2168 Shattuck Ave, Berkeley, Ca 94704, USA. Genomic biology (Review). *Cell* **100:** (1) 169.

Brewis IA. 1999. Univ Sheffield, Dept Mol Biol & Biotechnol, Sheffield S10 2TN, England. Proteomics in reproductive research: The potential importance of proteomics to research in reproduction. *Hum Reprod* **14:** (12) 2927.

Burnouf D, Miturski R*, Nagao M, Nakagama H, Nothisen M, Wagner J, Fuchs RPP. 2000. *Med Acad, Dept Gynecol Surg 2, Jaczewski St 8, PL-20290 Lublin, Poland. Molecular approach in cancer epidemiology: Early detection of carcinogen-induced mutations in a whole genome (Review). *Int J Mol Med* **5:** (1) 15.

Celis JE, Wolf H, Ostergaard M. 1999. Aarhus Univ, Dept Biochem Med, Ole Worms Alle, Bldg 170, DK-8000 Aarhus C, Denmark. Proteomic strategies in bladder cancer (Review). *IUBMB Life* **48:** (1) 19.

Colantuoni C, Purcell AE, Bouton CML, Pevsner J*. 2000. *Kennedy Krieger Inst, Dept Neurol, 707 Nth Broadway, Baltimore, Md 21205, USA. High throughput analysis of gene expression in the human brain (Mini-Review). *J Neurosci Res* **59:** (1) 1.

Debabov VG. 1999. State Res Ctr Gosniigenet, RU-113545 Moscow, Russia. Bacterial life outside laboratories. *Mol Biol-Engl Tr* **33:** (6) 950.

Dolnik V. 1999. Mol Dynam, 928 East Arques Ave, Sunnyvale, Ca 94086, USA. DNA sequencing by capillary electrophoresis (Review). *J Biochem Biophys Methods* **41:** (2-3) 103.

Durick K, Mendlein J, Xanthopoulos KC*. 1999. *Aurora Biosci Corp, San Diego, Ca 92121, USA. Hunting with traps: Genome-wide strategies for gene discovery and functional analysis. *Genome Res* **9:** (11) 1019.

Eddy SR. 1999. Washington Univ, Sch Med, Dept Genet, St Louis, Mo 63110, USA. Noncoding RNA genes. *Curr Opin Genet Dev* **9:** (6) 695.

Elskaya AV. 1999. Natl Acad Sci, Inst Mol Biol & Genet, UA-252143 Kiev, Ukraine. Regulation of protein synthesis in higher eukaryotes: Facts and hypotheses. *Mol Biol-Engl Tr* **33:** (6) 922.

Ferea TL, Brown PO. 1999. Stanford Univ, Dept Genet, Sch Med, Stanford, Ca 94305, USA. Observing the living genome. *Curr Opin Genet Dev* **9:** (6) 715.

Field D, Hood D, Moxon R. 1999. Univ Oxford, John Radcliffe Hosp, Dept Paediat, Mol Infect Dis Grp, Oxford OX3 9DS, England. Contribution of genomics to bacterial pathogenesis. *Curr Opin Genet Dev* **9:** (6) 700.

Gardner MJ. 1999. Inst Genom Res, 9712 Med Ctr Dr, Rockville, Md 20850, USA. The genome of the malaria parasite. *Curr Opin Genet Dev* **9:** (6) 704.

Gelfand MS, Mironov AA. 1999. State Res Ctr Gosniigenet, RU-113545 Moscow, Russia. Computational biology between decades. *Mol Biol-Engl Tr* **33:** (6) 854.

Gogarten JP, Olendzenski L. 1999. Univ Connecticut, Dept Mol & Cell Biol, 75 Nth Eagleville Rd, Storrs, Ct 06269, USA. Orthologs, paralogs and genome comparisons. *Curr Opin Genet Dev* **9:** (6) 630.

Gray MW. 1999. Dalhousie Univ, Canadian Inst Adv Res, Dept Biochem & Mol Biol, Program Evolutionary Biol, Halifax, Nova Scotia, Canada B3H 4H7. Evolution of organellar genomes. *Curr Opin Genet Dev* **9:** (6) 678.

Hernandez A, Evers BM*. 1999. *Univ Texas, Med Branch, Dept Surg, 301 Univ Blvd, Galveston, Tx 77555, USA. Functional genomics: Clinical effect and the evolving role of the surgeon. *Arch Surg* **134:** (11) 1209.

Hiltunen MO, Niemi M, Yla-Herttuala S. 1999. Univ Kuopio, AI Virtanen Inst, Dept Mol Med, POB 1627, FI-70211 Kuopio, Finland. Functional genomics and DNA array techniques in atherosclerosis research. *Curr Opin Lipidol* **10:** (6) 515.

Jacob HJ. 1999. Med Coll Wisconsin, Lab Genet Res, Milwaukee, Wi 53226, USA. Functional genomics and rat models. *Genome Res* **9:** (11) 1013.

Jungblut PR, Zimny-Arndt U, Zeindl-Eberhart E, Stulik J, Koupilova K, Pleissner KP, Otto A, Muller EC, Sokolowska-Kohler W, Grabher G, Stoffler G. 1999. Max Planck Inst Infect Biol, Prot Anal Unit, Monbijoustr 2, DE-10117 Berlin, Germany. Proteomics in human disease: Cancer, heart and infectious diseases - Review. *Electrophoresis* **20:** (10) 2100.

Kippert F, Hunt P. 2000. Univ Edinburgh, Inst Cell Anim & Populat Biol, Biol Timing Lab, Kings Bldg, Edinburgh EH9 3JN, Scotland. Ultradian clocks in eukaryotic microbes: from behavioural observation to

In order to keep subscribers up-to-date with the latest developments in their field, this current awareness service is provided by John Wiley & Sons and contains newly-published material on comparative and functional genomics. Each bibliography is divided into 16 sections. 1 Reviews & symposia; 2 General; 3 Large-scale sequencing and mapping; 4 Genome evolution; 5 Comparative genomics; 6 Gene families and regulons; 7 Pharmacogenomics; 8 Large-scale mutagenesis programmes; 9 Functional complementation; 10 Transcriptomics; 11 Proteomics; 12 Protein structural genomics; 13 Metabolomics; 14 Genomic approaches to development; 15 Technological advances; 16 Bioinformatics. Within each section, articles are listed in alphabetical order with respect to author. If, in the preceding period, no publications are located relevant to any one of these headings, that section will be omitted.

Copyright © 2000 John Wiley & Sons, Ltd.

functional genomics (Review). *Bioessays* **22:** (1) 16.

Kobayashi I, Nobusato A, Kobayashi-Takahashi N, Uchiyama I. 1999. Univ Tokyo, Inst Med Sci, 4-6-1 Shirokanedai, Tokyo 108 8639, Japan. Shaping the genome: Restriction-modification systems as mobile genetic elements. *Curr Opin Genet Dev* **9:** (6) 649.

Kondrashov AS. 1999. NIH/Natl Lib Med, Natl Ctr Biotechnol Informat, Bldg 38A, Bethesda, Md 20894, USA. Comparative genomics and evolutionary biology. *Curr Opin Genet Dev* **9:** (6) 624.

Kreitman M, Comeron JM. 1999. Univ Chicago, Dept Ecol & Evolut, 1101 East 57th St, Chicago, Il 60637, USA. Coding sequence evolution. *Curr Opin Genet Dev* **9:** (6) 637.

Lawrence J. 1999. Univ Pittsburgh, Dept Biol Sci, Pittsburgh, Pa 15260, USA. Selfish operons: The evolutionary impact of gene clustering in prokaryotes and eukaryotes. *Curr Opin Genet Dev* **9:** (6) 642.

Lim VI, Aglyamova GV. 1999. Russian Acad Sci, VA Engelhardt Mol Biol Inst, RU-117984 Moscow, Russia. The principles of formation of spatial structures of proteins and nucleic acids: Stereochemical modeling. *Mol Biol-Engl Tr* **33:** (6) 908.

Lyubomirskaya NV, Ilyin YV. 1999. Russian Acad Sci, VA Engelhardt Mol Biol Inst, RU-117984 Moscow, Russia. Eukaryotic mobile genetic elements: The past, the present, and the future. *Mol Biol-Engl Tr* **33:** (6) 846.

Marienfeld J, Unseld M, Brennicke A*. 1999. *Univ Ulm, Albert Einstein Allee, DE-89069 Ulm, Germany. The mitochondrial genome of *Arabidopsis* is composed of both native and immigrant information (Review). *Trends Plant Sci* **4:** (12) 495.

Mushegian A. 1999. Akkadix Corp, 11099 Nth Torrey Pines Rd, Suite 200, La Jolla, Ca 92037, USA. The minimal genome concept. *Curr Opin Genet Dev* **9:** (6) 709.

Nebert DW. 1999. Univ Cincinnati, Med Ctr, Ctr Environm Genet, Dept Environm Hlth, POB 670056, Cincinnati, Oh 45267, USA. Pharmacogenetics and pharmacogenomics: Why is this relevant to the clinical geneticist? (Mini-review). *Clin Genet* **56:** (4) 247.

Niimi M, Cannon RD, Monk BC. 1999. Univ Otago, Mol Microbiol Lab, Dept Oral Sci & Orthodont, Sch Dent, POB 647, Dunedin, New Zealand. *Candida albicans* pathogenicity: A proteomic perspective: Review. *Electrophoresis* **20:** (11) 2299.

Pang HM, Pavski V, Yeung ES*. 1999. *Iowa State Univ, US DOE, Ames Lab, Ames, Ia 50011, USA. DNA sequencing using 96-capillary array electrophoresis (Review). *J Biochem Biophys Methods* **41:** (2-3) 121.

Penny D, Poole A. 1999. Massey Univ, Inst Mol Biosci, POB 11 222, Palmerston North, New Zealand. The nature of the last universal common ancestor. *Curr Opin Genet Dev* **9:** (6) 672.

Porter CJ, Talbot CC, Cuticchia AJ*. 2000. *Hosp Sick Children, Res Inst, 555 Univ Ave, Toronto, Ontario, Canada M5G 1X8. Central mutation databases: A review. *Hum Mutat* **15:** (1) 36.

Praseuth D, Guieysse AL, Helene C. 1999. Museum Natl Hist Nat, CNRS, UMR 8646, INSERM, U201, Biophys Lab, 43 rue Cuvier, FR-75231 Paris 05, France. Triple helix formation and the antigene strategy for sequence-specific control of gene expression (Review). *Biochim Biophys Acta* **1489:** (1) 181.

Ptitsyn OB, Finkelstein AV, Dobson CM. 1999. Russian Acad Sci, Inst Prot Res, RU-142292 Pushchino, Russia. Protein folding: A bridge between physics and biology. *Mol Biol-Engl Tr* **33:** (6) 893.

Righetti PG, Gelfi C. 1999. Univ Verona, Dept Agr & Ind Biotechnol, Str Grazie, Ca Vignal, IT-37134 Verona, Italy. Capillary electrophoresis of DNA in the 20-500 bp range: Recent developments (Review). *J Biochem Biophys Methods* **41:** (2-3) 75.

Ryskov AP. 1999. Russian Acad Sci, Inst Gene Biol, RU-117989 Moscow, Russia. Multilocus DNA fingerprinting in the genetic studies of biodiversity. *Mol Biol-Engl Tr* **33:** (6) 880.

Skoogarev AV, Galzitskaya OV, Finkelstein AV. 1999. Russian Acad Sci, Inst Prot Res, RU-142292 Pushchino, Russia. Search for folding nuclei in three-dimensional protein structures. *Mol Biol-Engl Tr* **33:** (6) 897.

Smit AFA. 1999. Axys Pharmaceut Inc, 11099 Nth Torrey Pines Rd, Suite 160, La Jolla, Ca 92037, USA. Interspersed repeats and other mementos of transposable elements in mammalian genomes. *Curr Opin Genet Dev* **9:** (6) 657.

Souteyrand E. 1999. Ecole Cent Lyon, IFOS, CNRS, UMR 5621, 36 Ave Collongue, FR-69139 Ecully, France. DNA chips: From elaboration to application. *Analusis* **27:** (7) 639.

Subramanian P, Lakhotia SC*. 1999. *Banaras Hindu Univ, Dept Zool, Cytogenet Lab, IN-221005 Varanasi, Uttar Pradesh, India. Molecular rhythms that regulate rhythm genes in *Drosophila* (Review). *Curr Sci* **77:** (9) 1165.

Sverdlov ED. 1999. Russian Acad Sci, Inst Mol Genet, Kurchatov Sq, RU-123182 Moscow, Russia. The microcosm of the genome. *Mol Biol-Engl Tr* **33:** (6) 809.

Thiellement H, Bahrman N, Damerval C, Plomion C, Rossignol M, Santoni V, De Vienne D, Zivy M. 1999. Univ Geneva, Dept Biol Vegetal, 3 Pl Univ, CH-1211 Geneva 4, Switzerland. Proteomics for genetic and physiological studies in plants: Review. *Electrophoresis* **20:** (10) 2013.

Van Bogelen RA, Schiller EE, Thomas JD, Neidhardt FC. 1999. Warner Lambert Parke Davis, Parke Davis Pharmaceut Res Div, Dept Biol Mol, 2800 Plymouth Rd, Ann Arbor, Mi 48105, USA. Diagnosis of cellular states of microbial organisms using proteomics - Review. *Electrophoresis* **20:** (11) 2149.

Wang SS, Fernhoff PM, Hannon WH, Khoury MJ. 1999. Ctr Dis Control & Prevent, Off Genet & Dis Prevent, Natl Ctr Environm Hlth, Atlanta, Ga 30341, USA. Medium chain acyl-CoA dehydrogenase deficiency: Human genome epidemiology review (Review). *Genet Med* **1:** (7) 332.

Wilgenbus KK, Lichter P. 1999. Boehringer Ingelheim Austria, Dr Boehringergasse 5-11, AU-1121 Vienna, Austria. DNA chip technology *ante portas* (Review). *J Mol Med* **77:** (11) 761.

### 3 Large-scale sequencing and mapping

Albrecht JC. 2000. Univ Erlangen Nurnberg, Inst Klin & Mol Virol, Schlossgarten 4, DE-91054 Erlangen, Germany. Primary structure of the *Herpesvirus ateles* genome. *J Virol* **74:** (2) 1033.

Copenhaver GP, Nickel K, Kuromori T, Benito MI, Kaul S, Lin XY, Bevan M, Murphy G, Harris B, Parnell LD, McCombie WR, Martienssen RA, Marra M, Preuss D*. 1999. *Univ Chicago, Dept Mol Genet & Cell Biol, 1103 East 57 St, Chicago, Il 60637, USA. Genetic definition and sequence of *Arabidopsis* centromeres. *Science* **286:** (5449) 2468.

Jin Q, Zeng LY, Yang F, Li MX, Hou YD. 1999. State Key Lab Mol Virol & Genet Engn, CN-100052 Beijing, Peoples Rep China. The complete genomic sequence of egg drop syndrome virus strain AAV-2. *Sci China C Life Sci* **42:** (6) 607.

Koike H, Kawashima T, Suzuki M*. 1999. *AIST, NIBHT, CREST Ctr Struct Biol, 1-1 Higashi, Tsukuba, Ibaraki 305 0046, Japan. Enzymes identified using genomic DNA sequences suggest some atypical characteristics of *de novo* biosynthesis of purines in archaebacteria. *Proc Jpn Acad B* **75:** (8) 263.

Lesouef PN, Goldblatt J, Lynch NR. 1999. Children's Hosp Med Ctr, Dept Paediat, GPO Box D184, Perth, WA 6001, Australia. A genome screen and candidate gene studies in parasitized populations. *Clin Exp Allergy* **29:** (Suppl 4) 31.

Medigue C, Rose M, Viari A, Danchin A. 1999. Inst Pasteur Reg, FR-75724 Paris 15, France. Detecting and analyzing DNA sequencing errors: Toward a higher quality of the *Bacillus subtilis* genome sequence. *Genome Res* **9:** (11) 1116.

Copyright © 2000 John Wiley & Sons, Ltd. 

Surzycki SA, Belknap WR*. 2000. *USDA/ARS, Western Reg Res Cr, 800 Buchanan St, Albany, Ca 94710, USA. Repetitive-DNA elements are similarly distributed on *Caenorhabditis elegans* autosomes. *Proc Natl Acad Sci U S A* **97:** (1) 245.

Wan KL, Chong SP, Ng ST, Shirley MW, Tomley FM, Jangi MS. 1999. Univ Kebangsaan, Fac Sci & Technol, Sch Biosci & Biotechnol, Ctr Gene Anal & Technol, MY-43600 Bangui, Selangor, DE, Malaysia. A survey of genes in *Eimeria tenella* merozoites by EST sequencing. *Int J Parasitol* **29:** (12) 1885.

### 4 Genome evolution

Bennetzen JL. 2000. Purdue Univ, Dept Biol Sci, West Lafayette, In 47907, USA. Transposable element contributions to plant gene and genome evolution. *Plant Mol Biol* **42:** (1) 251.

De Oliveira PMC. 1999. Univ Fed Fluminense, Inst Fis, Avda Litoranea s/n, Boa Viagem, BR-24210-340 Niterol, RJ, Brazil. Studying DNA evolution through successive file editions. *Physica A* **273:** (1-2) 70.

Durbin ML, McCaig B, Clegg MT. 2000. Univ Calif, Dept Bot & Plant Sci, Riverside, Ca 92521, USA. Molecular evolution of the chalcone synthase multigene family in the morning glory genome. *Plant Mol Biol* **42:** (1) 79.

Ge S, Sang T, Lu BR, Hong DY. 1999. Michigan State Univ, Dept Bot & Plant Pathol, East Lansing, Mi 48824, USA. Phylogeny of rice genomes with emphasis on origins of allotetraploid species. *Proc Natl Acad Sci U S A* **96:** (25) 14400.

Hannoun C, Horal P, Lindh M*. 2000. *Gothenburg Univ, Dept Clin Virol, Guldhedsgatan 108, SE-41346 Gothenburg, Sweden. Long-term mutation rates in the hepatitis B virus genome. *J Gen Virol* **81:** (1) 75.

James TY, Porter D, Hamrick JL, Vilgalys R. 1999. Duke Univ, Dept Bot, Box 90338, Durham, NC 27708, USA. Evidence for limited intercontinental gene flow in the cosmopolitan mushroom, *Schizophyllum commune*. *Evolution* **53:** (6) 1665.

Kappen C. 2000. Mayo Clin Scottsdale, SC Johnson Med Res Ctr, 13400 East Shea Blvd, Scottsdale, Az 85259, USA. The homeodomain: An ancient evolutionary motif of animals and plants. *Comput Chem* **24:** (1) 95.

Kowalczuk M, Gierlik A, Mackiewicz P, Cebrat S, Dudek MR*. 1999. *Univ Wroclaw, Inst Microbiol, ul Przybyszewskiego 63-77, PL-54148 Wroclaw, Poland. Optimization of gene sequences under constant mutational pressure and selection. *Physica A* **273:** (1-2) 116.

Lobry JR. 1999. Univ Lyon 1, CNRS UMR 5558, Lab BBE, 43 Blvd 11 Novembre 1918, FR-69622 Villeurbanne, France. A nice wrong model for the evolution of DNA base frequencies. *Physica A* **273:** (1-2) 99.

Mackiewicz P, Gierlik A, Kowalczuk M, Szczepanik D, Dudek MR, Cebrat S*. 1999. *Univ Wroclaw, Inst Microbiol, Dept Genet, ul Przybyszewskiego 63-77, PL-51148 Wroclaw, Poland. Mechanisms generating long-range correlation in nucleotide composition of the *Borrelia burgdoferi* genome. *Physica A* **273:** (1-2) 103.

Majumdar S, Gupta SK, Sundararajan VS, Ghosh TC*. 1999. *Bose Inst, Distributed Informat Ctr, P1/12 CIT Scheme VII M, IN-700054 Calcutta, India. Compositional correlation studies among the three different codon positions in 12 bacterial genomes. *Biochem Biophys Res Commun* **266:** (1) 66.

Noguchi S, Tajima T, Yamamoto Y, Ohno T, Kubo T. 1999. Japan Tobacco Inc, Leaf Tobacco Res Lab, 1900 Idei, Oyama, Tochigi 323 0808, Japan. Deletion of a large genomic segment in tobacco varieties that are resistant to potato virus Y (PVY). *Mol Gen Genet* **262:** (4-5) 822.

Parkinson CL, Adams KL, Palmer JD*. 1999. *Indiana Univ, Dept Biol, Jordan Hall, 1001 East 3rd St, Bloomington, In 47405, USA. Multigene analyses identify the three earliest lineages of extant flowering plants. *Curr Biol* **9:** (24) 1485.

Rosche WA, Foster PL*. 2000. *Indiana Univ, Dept Biol, 1001 East 3rd St, Bloomington, In 47405, USA. Determining mutation rates in bacterial populations. *Methods* **20:** (1) 4.

Salyers AA, Bonheyo G, Shoemaker NB. 2000. Univ Illinois, Dept Microbiol, 601 Sth Goodwin Ave, Urbana, Il 61801, USA. Starting a new genetic system: Lessons from *Bacteroides*. *Methods* **20:** (1) 35.

Schmid KJ, Nigro L, Aquadro CF, Tautz D. 1999. Cornell Univ, Dept Mol Biol & Genet, Genet & Dev Sect, 403 Biotechnol Bldg, Ithaca, NY 14853, USA. Large number of replacement polymorphisms in rapidly evolving genes of *Drosophila*: Implications for genome-wide surveys of DNA polymorphism. *Genetics* **153:** (4) 1717.

Stuyver L, De Gendt S, Van Geyt C*, Zoulim F, Fried M, Schinazi RF, Rossau R. 2000. *Innogenet NV, Ind Pk 7, Box 4, BE-9052 Ghent, Belgium. A new genotype of hepatitis B virus: Complete genome and phylogenetic relatedness. *J Gen Virol* **81:** (1) 67.

Sverdlov ED. 1999. Russian Acad Sci, Inst Mol Genet, ul Kurchatova 46, RU-123182 Moscow, Russia. Retroviral regulators of gene expression in human genome as possible factors of its evolution (Russian, English Abstract). *Bioorg Khim* **25:** (11) 821.

Van der Drift P, Chan A, Zehetner G, Westerveld A, Versteeg R*. 1999. *Univ Amsterdam, Acad Med Ctr, Dept Human Genet, POB 22700, NL-1100 DE Amsterdam, The Netherlands. Multiple MSP pseudogenes in a local repeat cluster on 1p36.2: An expanding genomic graveyard? *Genomics* **62:** (1) 74.

Wendel JF. 2000. Iowa State Univ Sci & Technol, Dept Bot, Ames, Ia 50011, USA. Genome evolution in polyploids. *Plant Mol Biol* **42:** (1) 225.

Yang J, Bogerd HP, Peng S, Wiegand H, Truant R, Cullen BR*. 1999. *Duke Univ, Med Ctr, Howard Hughes Med Inst, Box 3025, Durham, NC 27710, USA. An ancient family of human endogenous retroviruses encodes a functional homolog of the HIV-1 Rev protein. *Proc Natl Acad Sci U S A* **96:** (23) 13404.

### 5 Comparative genomics

Barakat A, Han DT, Benslimane AA, Rode A, Bernardi G*. 1999. *Inst Jacques Monod, Genet Mol Lab, 2 Pl Jussieu, FR-75005 Paris, France. The gene distribution in the genomes of pea, tomato and date palm. *FEBS Lett* **463:** (1-2) 139.

Bogush ML, Velikodvorskaya TV, Lebedev YB, Nikolaev LG, Lukyanov SA, Fradkov AF, Pliyev BK, Boichenko MN, Usatova GN, Vorobiev AA, Andersen GL, Sverdlov ED*. 1999. *Russian Acad Sci, Shemyakin Ovchinnikov Inst Bio-organ Chem, RU-117871 Moscow, Russia. Identification and localization of differences between *Escherichia coli* and *Salmonella typhimurium* genomes by suppressive subtractive hybridization. *Mol Gen Genet* **262:** (4-5) 721.

Caetano AR, Shiue YL, Lyons LA, O'Brien SJ, Laughlin TF, Bowling AT, Murray JD*. 1999. *Univ Calif Davis, Dept Anim Sci, Livermore, Ca 95616, USA. A comparative gene map of the horse (*Equus caballus*). *Genome Res* **9:** (12) 1239.

Fukunishi Y, Suzuki H, Yoshino M, Konno H, Hayahizaki Y. 1999. Inst Phys & Chem Res, Tsukuba Lifesci Ctr, Genome Sci Lab, 3-1-1 Koyadai, Tsukuba, Ibaraki 305 0074, Japan. Prediction of human cDNA from its homologous mouse full-length cDNA and human shotgun database. *FEBS Lett* **464:** (3) 129.

Groenen MAM, Croojimans RPMA, Dijkhof RJM, Van der Poel JJ. 1999. Wageningen Univ Agr, Wageningen Inst Anim Sci, Anim Breeding & Genet Grp, POB 338, NL-6700 AH Wageningen, The Netherlands. Extending the chicken-human comparative map by placing 15 genes on the chicken linkage map. *Anim Genet* **30:** (6) 418.

Hu J, Wills M, Baker BA, Perlman EJ*. 2000. *Johns Hopkins Univ Hosp, Pathol Bldg, Room 612, 600 Nth Wolfe St, Baltimore, Md 21287, USA. Comparative genomic hybridization analysis of hepatoblastomas. *Gene Chromosomes Cancer* **27:** (2) 196.

Copyright © 2000 John Wiley & Sons, Ltd.

Jay V, Squire J, Bayani J, Alkhani AM, Rutka JT, Zielenska M. 1999. Univ Toronto, Hosp Sick Children, Div Pathol, 555 Univ Ave, Toronto, Ontario, Canada M5G 1X8. Oncogene amplification in medulloblastoma: Analysis of a case by comparative genomic hybridization and fluorescence *in situ* hybridization. *Pathology* **31:** (4) 337.

Jurado LAP, Wang YK, Francke U, Cruces J. 1999. Hosp Univ La Paz, Serv Genet, Paseo Castellana 261, ES-28046 Madrid, Spain. TBL2, a novel transducin family member in the WBS deletion: Characterization of the complete sequence, genomic structure, transcriptional variants and the mouse ortholog. *Cytogenet Cell Genet* **86:** (3-4) 277.

Karch H, Schmidt H, Janetzki-Mittmann C, Scheef J, Kroger M*. 1999. *Univ Wurzburg, Inst Hyg & Mikrobiol, Josef Schneider Str 2, GE-98070 Wurzburg, Germany. Shiga toxins even when different are encoded at identical positions in the genomes of related temperate bacteriophages. *Mol Gen Genet* **262:** (4-5) 600.

Murphy WJ, Sun S, Chen ZQ, Pecon-Slattery J, O'Brien SJ. 1999. NCI, Frederick Canc Res & Dev Ctr, Lab Genom Divers, Frederick, Md 21702, USA. Extensive conservation of sex chromosome organization between cat and human revealed by parallel radiation hybrid mapping. *Genome Res* **9:** (12) 1223.

Plucienniczak G, Plucienniczak A*. 1999. *Inst Biotechnol & Antibiot, Staroscinska 5, PL-02516 Warsaw, Poland. Fragments of LINE-1 retrotransposons flanked by inverted telomeric repeats are present in the bovine genome. Homology with human LINE-1 elements. *Acta Biochim Pol* **46:** (4) 873.

Radda GK. 1999. Univ Oxford, Dept Biochem, Sth Parks Rd, Oxford OX1 3QU, England. Of mice and men: From early NMR studies of the heart to physiological genomics. *Biochem Biophys Res Commun* **266:** (3) 723.

Sawant SV, Singh PK, Gupta SK, Madnala R, Tuli R*. 1999. *Natl Bot Res Inst, Rana Pratap Marg, IN-226001 Lucknow, India. Conserved nucleotide sequences in highly expressed genes in plants. *J Genet* **78:** (2) 123.

Serakinci N, Krejci K, Koch J*. 1999. *Danish Canc Soc, Dept Cytogenet, Tage Hansens Gade 2, DK-2800 Lyngby C, Denmark. Telomeric repeat organization: A comparative *in situ* study between man and rodent. *Cytogenet Cell Genet* **86:** (3-4) 204.

Takahashi C, Marshall JA, Bennett MD, Leitch IJ*. 1999. *Royal Bot Gardens, Jodrell Lab, Richmond TW9 3AB, England. Genomic relationships between maize and its wild relatives. *Genome* **42:** (6) 1201.

Wiltshire T, Pletcher M, Cole SE, Villanueva M, Birren B, Lehoczky J, Dewar K, Reeves RH*. 1999. *Johns Hopkins Univ, Sch Med, Dept Physiol, Baltimore, Md 21205, USA. Perfect conserved linkage across the entire mouse chromosome 10 region homologous to human chromosome 21. *Genome Res* **9:** (12) 1214.

### 6 Gene families and regulons

Belanger M, Burrows LL, Lam JS*. 1999. *Univ Guelph, Dept Microbiol, Guelph, Ontario, Canada N1G 2W1. Functional analysis of genes responsible for the synthesis of the B-band O antigen of *Pseudomonas aeruginosa* serotype O6 lipopolysaccharide. *Microbiology* **145:** (12) 3505.

Danchin A. 1999. Inst Pasteur, CNRS, URA 1129, 28 rue Dr Roux, FR-75724 Paris 15, France. From function to sequence, an integrated view of the genome texts. *Physica A* **273:** (1-2) 92.

Fujinaga K, Taniguchi Y, Sun YZ, Katayama S, Minami J, Matsushita O, Okabe A*. 1999. *Kagawa Med Univ, Fac Med, Dept Microbiol, Miki, Kagawa 761 0793, Japan. Analysis of genes involved in nitrate reduction in *Clostridium perfringens*. *Microbiology* **145:** (12) 3377.

Klassen G, Pedrosa FO, Souza EM, Yates MG, Rigo LU*. 1999. *Univ Fed Parana, Dept Bioquim, CP 19046, BR-81531-990 Curitiba, Parana, Brazil. Sequencing and functional analysis of the *nifENXorf1orf2* gene cluster of *Herbaspirillum seropedicae*. *FEMS Microbiol Lett* **181:** (1) 165.

McCann RO, Craig SW*. 1999. *Johns Hopkins Univ, Sch Med, Dept Biol Chem, 725 Nth Wolfe St, Baltimore, Md 21205, USA. Functional genomic analysis reveals the utility of the I/LWEQ module as a predictor of protein-actin interaction. *Biochem Biophys Res Commun* **266:** (1) 135.

Nishida H, Nishiyama M*, Kobashi N, Kosuge T, Hoshino T, Yamane H. 1999. *Univ Tokyo, Biotechnol Res Ctr, Tokyo 113 8657, Japan. A prokaryotic gene cluster involved in synthesis of lysine through the amino adipate pathway: A key to the evolution of amino acid biosynthesis. *Genome Res* **9:** (12) 1175.

Ouspenski II, Elledge SJ, Brinkley BR. 1999. Baylor Coll Med, Dept Cell Biol, 1 Baylor Plaza, Houston, Tx 77030, USA. New yeast genes important for chromosome integrity and segregation identified by dosage effects on genome stability. *Nucleic Acids Res* **27:** (15) 3001.

Reizer J, Bachem S, Reizer A, Arnaud M, Saier MH, Stulke J*. 1999. *Univ Erlangen Nurnberg, Lehrstuhl Mikrobiol, Inst Mikrobiol Biochem & Genet, Staudtstr 5, DE-91058 Erlangen, Germany. Novel phosphotransferase system genes revealed by genome analysis: The complete complement of PTS proteins encoded within the genome of *Bacillus subtilis*. *Microbiology* **145:** (12) 3419.

Suriyapperuma SP, Lozovatsky L, Ciciotte SL, Peters LL, Gilligan DM*. 2000. *Yale Univ, Sch Med, 333 Cedar St, New Haven, Ct 06510, USA. The mouse adducin gene family: Alternative splicing and chromosomal localization. *Mamm Genome* **11:** (1) 16.

### 7 Pharmacogenomics

Collins A, Lonjou C, Morton NE*. 1999. *Univ Southampton, Southampton Gen Hosp, Tremona Rd, Southampton SO16 6YD, England. Genetic epidemiology of single-nucleotide polymorphisms. *Proc Natl Acad Sci U S A* **96:** (26) 15173.

Gray CP, Keck W. 1999. F Hoffmann La Roche & Co Ltd, Pharma Res Preclin, CH-4070 Basel, Switzerland. Bacterial targets and antibiotics: Genome-based drug discovery. *Cell Mol Life Sci* **56:** (9-10) 779.

Nickerson T, Pollak M*. 1999. *Lady Davis Inst Med Res, 3755 Cote St, Catherine Rd, Montreal, Quebec, Canada H3T 1E2. Bicalutamide (Casodex)-induced prostate regression involves increased expression of genes encoding insulin-like growth factor binding proteins. *Urology* **54:** (6) 1120.

Vogt PK, Aoki M, Bottoli I, Chang HW, Fu SL, Hecht A, Iacovoni JS, Jiang BH, Kruse U. 1999. Scripps Clin & Res Inst, Dept Mol & Expt Med, 10550 Nth Torrey Pines Rd, La Jolla, Ca 92037, USA. A random walk in oncogene space: The quest for targets. *Cell Growth Differ* **10:** (12) 777.

### 8 Large-scale mutagenesis programmes

Berry AM, Paton JC*. 2000. *Women's & Children's Hosp, Mol Microbiol Unit, Adelaide, SA 5006, Australia. Additive attenuation of virulence of *Streptococcus pneumoniae* by mutation of the genes encoding pneumolysin and other putative pneumococcal virulence proteins. *Infect Immun* **68:** (1) 133.

Entian KD, Schuster T, Hegemann JH, Becher D, Feldmann H, Guldener U, Gotz R, Hansen M, Hollenberg CP, Jansen G, Kramer W, Klein S, Kotler P, Kricke J, Launhardt H, Mannhaupt G, Maierl A, Meyer P, Mewes W, Munder T, Niedenthal RK, Ramezani-Rad M, Rohmer A, Romer A, Rose M, Schafer B, Siegler ML, Vetter J, Wilhelm N, Wolf K, Zimmerman FK, Zollner A, Hinnen A. 1999. Univ Frankfurt, Inst Mikrobiol, Marie Curie Str 9, DE-60439 Frankfurt, Germany. Functional analysis of 150 deletion mutants in *Saccharomyces cerevisiae* by a systematic approach. *Mol Gen Genet* **262:** (4-5) 683.

Krysan PJ, Young JC, Sussman MR*. 1999. *Univ Wisconsin, Ctr Bio-

Copyright © 2000 John Wiley & Sons, Ltd. 

technol, 425 Henry Mall, Madison, Wi 53706, USA. T-DNA as an insertional mutagen in *Arabidopsis*. *Plant Cell* **11:** (12) 2283.

Parinov S, Sevugan M, Ye D, Yang WC, Kumaran M, Sundaresan V*. 1999. *Natl Univ Singapore, Inst Mol Agrobiol, 1 Res Link, SG-117604 Singapore, Singapore. Analysis of flanking sequences from dissociation insertion lines: A database for reverse genetics in *Arabidopsis*. *Plant Cell* **11:** (12) 2263.

Parish T, Gordhan BG, McAdam RA, Duncan K, Mizrahi V, Stoker NG*. 1999. *Univ London, London Sch Hyg & Trop Med, Dept Infect & Trop Dis, Keppel St, London WC1E 7HT, England. Production of mutants in amino acid biosynthesis genes of *Mycobacterium tuberculosis* by homologous recombination. *Microbiology* **145:** (12) 3487.

Payne CM, Mullins LJ, Mullins JJ. 1999. Univ Edinburgh, Dept Vet Pathol, Summerhall Sq, Edinburgh EH9 1QH, Scotland. Manipulating large genomic clones *via in vivo* recombination in bacteria. *J Hum Hypertens* **13:** (12) 845.

Rathkolb B, Krebs O, Balling R, De Angelis MH, Wolf E. 1999. Univ Munich, Gene Ctr, Inst Mol Anim Breeding, Feodor Lynen Str 25, DE-81377 Munich, Germany. Discovery and functional characterisation of new genes by large scale ENU mutagenesis in mice. *Arch Tierzucht* **42: (**Spec Iss**)** 74.

Ross-MacDonald P, Coelho PSR, Roemer T, Agarwal S, Kumar A, Jansen R, Cheung KH, Sheehan A, Symoniatis D, Umansky L, Heldtman M, Nelson FK, Iwasaki H, Hager K, Gerstein M, Miller P, Roeder GS, Snyder M*. 1999. *Yale Univ, Dept Mol Cellular & Dev Biol, POB 208103, New Haven, Ct 06520, USA. Large-scale analysis of the yeast genome by transposon tagging and gene disruption. *Nature* **402:** (6760) 413.

Su H, Wang XH, Bradley A*. 2000. *Baylor Coll Med, Program Dev Biol, Houston, Tx 77030, USA. Nested chromosomal deletions induced with retroviral vectors in mice. *Nat Genet* **24:** (1) 92.

Wilson RB, Davis D, Enloe BM, Mitchell AP*. 2000. *Columbia Univ, Dept Microbiol, 701 West 168th St, New York, NY 10032, USA. A recyclable *Candida albicans* URA3 cassette for PCR product-directed gene disruptions. *Yeast* **16:** (1) 65.

### 10 Transcriptomics

Bellen HJ. 1999. Baylor Coll Med, Hughes Med Inst, Dept Mol & Human Genet, Program Dev Biol, Houston, Tx 77030, USA. Ten years of enhancer detection: Lessons from the fly. *Plant Cell* **11:** (12) 2271.

Bowler LD, Hubank M, Spratt BG. 1999. Univ Sussex, Sch Biol Sci, Brighton BN1 9QG, England. Representational difference analysis of cDNA for the detection of differential gene expression in bacteria: Development using a model of iron-regulated gene expression in *Neisseria meningitidis*. *Microbiology* **145:** (12) 3529.

Heyer LJ, Kruglyak S*, Yooseph S. 1999. *Univ Sthn Calif, Dept Math, Los Angeles, Ca 90089, USA. Exploring expression data: Identification and analysis of coexpressed genes. *Genome Res* **9:** (11) 1106.

Johannes G, Carter MS, Eisen MB, Brown PO*, Sarnow P. 1999. *Stanford Univ, Sch Med, Dept Microbiol & Immunol, Stanford, Ca 94305, USA. Identification of eukaryotic mRNAs that are translated at reduced cap binding complex eIF4F concentrations using a cDNA microarray. *Proc Natl Acad Sci U S A* **96:** (23) 13118.

Jung US, Levin DE*. 1999. *Johns Hopkins Univ, Sch Publ Hlth, Dept Biochem & Mol Biol, 615 Nth Wolfe St, Baltimore, Md 21205, USA. Genome-wide analysis of gene expression regulated by the yeast cell wall integrity signalling pathway. *Mol Microbiol* **34:** (5) 1049.

Kawamoto S, Ohnishi T, Kita H, Chisaka O, Okubo K*. 1999. *Osaka Univ, Inst Mol & Cellular Biol, 1-3 Yamada Oka, Suita, Osaka 565, Japan. Expression profiling of iAFLP: A PCR-based method for genome wide gene expression profiling. *Genome Res* **9:** (12) 1305.

Mochii M, Yoshida S, Morita K, Kohara Y, Ueno N. 1999. Natl Inst Basic Biol, Dept Dev Biol, Okazaki, Aichi 444 8585, Japan. Identification of transforming growth factor-β-regulated genes in *Caenorhabditis elegans* by differential hybridization of arrayed cDNAs. *Proc Natl Acad Sci U S A* **96:** (26) 15020.

Moradpour D, Blum HE. 1999. Univ Freiburg, Med Klin, Hugstetter Str 55, DE-7800 Freiburg, Germany. DNA chip-technology (German). *Dtsch Med Wochenschr* **124:** (46) 1395.

Pietu G, Eveno E, Soury-Segurens B, Fayein NA, Mariage-Samson R, Matingou C, Leroy E, Dechesne C, Krieger S, Ansorge W, Reguigne-Arnould I, Cox D, Dehejia A, Polymerpoulos MH, Devignes MD, Auffray C. 1999. Genexpress, CNRS ERS 1984, FR-94801 Villejuif, France. The Genexpress IMAGE knowledge base of the human muscle transcriptome: A resource of structural, functional, and positional candidate genes for muscle physiology and pathologies. *Genome Res* **9:** (12) 1313.

Piunno PAE, Watterson J, Wust CC, Krull UJ*. 1999. *Univ Toronto, Dept Chem, Chem Sensors Grp, 3359 Mississauga Rd Nth, Mississauga, Ontario, Canada L5L 1C6. Considerations for the quantitative transduction of hybridization of immobilized DNA. *Anal Chim Acta* **400:** 73.

Roberts CJ, Nelson B, Marton MJ, Stoughton R, Meyer MR, Bennett HA, He YDD, Dai HY, Walker WL, Hughes TR, Tyers M, Boone C*, Friend SH. 2000. *Queen's Univ, Biol Dept, Kingston, Ontario, Canada K7L 3N6. Signaling and circuitry of multiple MAPK pathways revealed by a matrix of global gene expression profiles. *Science* **287:** (5454) 873.

Schalkwyk LC, Himmelbauer H, Lehrach H. 1999. Max Planck Inst Mol Genet, Ihnestr 73, DE-14195 Berlin, Germany. Toward the mammalian transcript map. *Arch Tierzucht* **42: (**Spec Iss**)** 67.

Syn CKC, Teo WL, Swarup S*. 1999. *Natl Univ Singapore, Dept Biol Sci, Lower Kent Ridge, SG-Singapore, Rep Singapore. Three-detergent method for the extraction of RNA from several bacteria. *Biotechniques* **27:** (6) 1140.

Ter Linde JJM, Liang H, Davis RW, Steensma HY*, Van Dijken JP, Pronk JT. 1999. *Clusius Lab, Wassenaarseweg 64, NL-2333 AL Leiden, The Netherlands. Genome-wide transcriptional analysis of aerobic and anaerobic chemostat cultures of *Saccharomyces cerevisiae*. *J Bacteriol* **181:** (24) 7409.

Vinnemeier J, Hagemann M*. 1999. *Univ Rostock, Fachbereich Biol, Doberaner Str 143, DE-18051 Rostock, Germany. Identification of salt-regulated genes in the genome of the cyanobacterium *Synechocystis* sp strain PCC 6803 by subtractive RNA hybridization. *Arch Microbiol* **172:** (6) 377.

Virlon B, Cheval L, Buhler JM, Billon E, Doucet A, Elalouf JM*. 1999. *CEA Saclay, Dept Biol Cellulaire & Mol, Serv Biol Cellulaire, CNRS, Unite Rech Associee 1859, FR-91191 Gif-sur-Yvettes, France. Serial microanalysis of renal transcriptomes. *Proc Natl Acad Sci U S A* **96:** (26) 15286.

Walker MG, Volkmuth W, Sprinzak E, Hodgson D, Klingler T. 1999. Incyte Pharmaceut, Palo Alto, Ca 94304, USA. Prediction of gene function by genome-scale expression analysis: Prostate cancer-associated genes. *Genome Res* **9:** (12) 1198.

Wildt S, Deuschle U*. 1999. *Hoffmann La Roche AG, Div Pharma, Preclin CNS Res & Genetechnol, CH-4002 Basel, Switzerland. *cobA*, a red fluorescent transcriptional reporter for *Escherichia coli*, yeast, and mammalian cells. *Nat Biotechnol* **17:** (12) 1175.

Wyrick JJ, Holstege FCP, Jennings EG, Causton HC, Shore D, Grunstein M, Lander ES, Young RA*. 1999. *MIT, Dept Biol, 77 Massachusetts Ave, Cambridge, Ma 02139, USA. Chromosomal landscape of nucleosome-dependent gene expression and silencing in yeast. *Nature* **402:** (6760) 418.

### 11 Proteomics

Adams P, Fowler R, Howell G, Kinsella N, Skipp P, Coote P, O'Connor

Copyright © 2000 John Wiley & Sons, Ltd. 

CD*. 1999. *Univ Southampton, Sch Biol Sci, Dept Biochem, Div Biochem & Mol Biol, Bassett Crescent East, Southampton SO16 7PX, England. Defining protease specificity with proteomics: A protease with a dibasic amino acid recognition motif is regulated by a two-component signal transduction system in *Salmonella*. *Electrophoresis* **20:** (11) 2241.

Beranova-Giorgianni S, Desiderio DM*. 2000. *Univ Tennessee, 847 Monroe Ave, Room 117, Memphis, Tn 38163, USA. Mass spectrometry of the human pituitary proteome: Identification of selected proteins. *Rapid Commun Mass Spectrom* **14:** (3) 161.

Bernhardt J, Buttner K, Scharf C, Hecker M*. 1999. *Univ Greifswald, Inst Mikrobiol & Mol Biol, Friedrich Ludwig Jahn Str 15, DE-17487 Greifswald, Germany. Dual channel imaging of two-dimensional electropherograms in *Bacillus subtilis*. *Electrophoresis* **20:** (11) 2225.

Cash P, Argo E, Ford L, Lawrie L, McKenzie H. 1999. Univ Aberdeen, Dept Med Microbiol, Polwarth Bldg, Aberdeen AB25 2ZD, Scotland. A proteomic analysis of erythromycin resistance in *Streptococcus pneumoniae*. *Electrophoresis* **20:** (11) 2259.

Dalluge JJ, Reddy P. 2000. NIST, 100 Bureau Dr, Stop 8392, Gaithersburg, Md 20899, USA. Identification of proteins in cell-free extracts using liquid chromatography-electrospray ionization mass spectrometry. *Biotechniques* **28:** (1) 156.

Edgar PF, Douglas JE, Knight C, Cooper GJS, Faull RLM, Kydd R. 1999. Univ Auckland, Sch Biol Sci, Thomas Bldg, 4th Floor, 3A Symonds St, Private Bag 9, Auckland, New Zealand. Proteome map of the human hippocampus. *Hippocampus* **9:** (6) 644.

Feng BB, Smith RD*. 2000. *Pacific NW Labs, Environm Mol Sci Lab, POB 999, Richland, Wa 99352, USA. A simple nanoelectrospray arrangement with controllable flowrate for mass analysis of submicroliter protein samples. *J Am Soc Mass Spectrom* **11:** (1) 94.

Frisa PS, Lanford RE, Jacobberger JW*. 2000. *Case Western Reserve Univ, Ctr Canc Res, 10900 Euclid Ave, Cleveland, Oh 44106, USA. Molecular quantification of cell cycle-related gene expression at the protein level. *Cytometry* **39:** (1) 79.

Hirose I, Sano K, Shioda I, Kumano M, Nakamura K, Yamane K*. 2000. *Univ Tsukuba, Inst Biol Sci, Ibaraki, Osaka 305, Japan. Proteome analysis of *Bacillus subtilis* extracellular proteins: A two-dimensional protein electrophoretic study. *Microbiology* **146:** (1) 65.

Kanitz MH, Witzmann FA, Zhu H, Fultz CD, Skaggs S, Moorman WJ, Savage RE. 1999. NIOSH, Div Biomed & Behav Sci, Expt Toxicol Branch, 4676 Columbia Pkwy, MS-C-23, Cincinnati, Oh 45226, USA. Alterations in rabbit kidney protein expression following lead exposure as analyzed by two-dimensional gel electrophoresis. *Electrophoresis* **20:** (14) 2977.

Manson MD. 2000. Texas A&M Univ, Dept Biol, College Station, Tx 77843, USA. Allele-specific suppression as a tool to study protein-protein interactions in bacteria. *Methods* **20:** (1) 18.

Mitaku S, Ono M, Hirokawa T, Boon-Chieng S, Sonoyama M. 1999. Tokyo Univ Agr & Technol, Dept Biotechnol, Tokyo 184 8588, Japan. Proportion of membrane proteins in proteomes of 15 single-cell organisms analyzed by the SOSUI prediction system. *Biophys Chem* **82:** (2-3) 165.

Mollenkopf HJ, Jungblut PR, Raupach B, Mattow J, Lamer S, Zimny-Arndt U, Schaible UE, Kaufmann SHE. 1999. Max Planck Inst Infect Biol, Dept Immunol, Monbijoustr 2, DE-10117 Berlin, Germany. A dynamic two-dimensional polyacrylamide gel electrophoresis database: The mycobacterial proteome *via* Internet. *Electrophoresis* **20:** (11) 2172.

Perrot M, Sagliocco F, Mini T, Monribot C, Schneider U, Shevchenko A, Mann M, Jeno P, Boucherie H*. 1999. *UPR CNRS 9026, Inst Biochim & Genet Cellulaires, 1 rue Camille St Saens, FR-33077 Bordeaux, France. Two-dimensional gel protein database of *Saccharomyces cerevisiae* (update 1999). *Electrophoresis* **20:** (11) 2280.

Phan-Thanh L, Mahouin F. 1999. INRA, Pathol Infect & Immunol Lab, FR-37380 Nouzilly, France. A proteomic approach to study the acid response in *Listeria monocytogenes*. *Electrophoresis* **20:** (11) 2214.

Quadroni M, James P, Dainese-Hatt P, Kertesz MA*. 1999. *ETH Zentrum, Swiss Fed Inst Technol, Inst Mikrobiol, CH-8092 Zurich, Switzerland. Proteome mapping, mass spectrometric sequencing and reverse transcription-PCR for characterization of the sulfate starvation-induced response in *Pseudomonas aeruginosa* PA01. *Eur J Biochem* **266:** (3) 986.

Rabilloud T. 2000. CEA, BECP, DBMS, 17 rue Martyrs, FR-38054 Grenoble, France. Detecting proteins separated by 2-D gel electrophoresis. *Anal Chem* **72:** (1) 48A.

Sanchez-Campillo M, Bini L, Comanducci R, Raggiaschi R, Marzocchi B, Pallini V, Ratti G*. 1999. *IRIS, Res Ctr, 1 via Fiorentina, IT-53100 Siena, Italy. Identification of immunoreactive proteins of *Chlamydia trachomatis* by Western blot analysis of a two-dimensional electrophoresis map with patient sera. *Electrophoresis* **20:** (11) 2269.

Sazuka T, Yamaguchi M, Ohara O*. 1999. *1532-3 Yana, Chiba 292 0812, Japan. Cyano2Dbase updated: Linkage of 234 protein spots to corresponding genes through N-terminal microsequencing. *Electrophoresis* **20:** (11) 2160.

Sherrier DJ, Prime TA, Dupree P*. 1999. *Univ Cambridge, Dept Biochem, Tennis Court Rd, Cambridge CB2 1QW, England. Glycosylphosphatidylinositol-anchored cell-surface proteins from *Arabidopsis*. *Electrophoresis* **20:** (10) 2027.

Sinha P, Hutter G, Kottgen E, Dietel M, Schadendorf D, Lage H. 1999. Humboldt Univ, Fak Med, Klinikum Charite, Inst Lab Med & Pathobiochem, Schumannstr 20-21, Berlin, Germany. Increased expression of epidermal fatty acid binding protein, cofilin, and 14-3-3-δ(stratifin) detected by two-dimensional gel electrophoresis, mass spectrometry and microsequencing of drug-resistant human adenocarcinoma of the pancreas. *Electrophoresis* **20:** (14) 2952.

Sinha P, Hutter G, Kottgen E, Dietel M, Schadendorf D, Lage H. 1999. Address as above. Search for novel proteins involved in the development of chemoresistance in colorectal cancer and fibrosarcoma cells *in vitro* using two-dimensional electrophoresis, mass spectrometry and microsequencing. *Electrophoresis* **20:** (14) 2961.

Vasseur C, Labadie J, Hebraud M. 1999. INRA, Unite Rech Viande, Equipe Microbiol, FR-63122 St Genes Champanelle, France. Differential protein expression by *Pseudomonas fragi* submitted to various stresses. *Electrophoresis* **20:** (11) 2204.

Veenstra TD, Martinovic S, Anderson GA, Pasa-Tolic L, Smith RD*. 2000. *Pacific NW Labs, Environm & Mol Sci Lab, POB 999, Richland, Wa 99352, USA. Proteome analysis using selective incorporation of isotopically labeled amino acids. *J Am Soc Mass Spectrom* **11:** (1) 78.

Wasinger VC, Urquhart BL, Humphrey-Smith I. 1999. Garvan Inst Med Res, 384 Victoria St, Darlinghurst, NSW 2010, Australia. Cross-species characterisation of abundantly expressed *Ochrobactrum anthropi* gene products. *Electrophoresis* **20:** (11) 2196.

Yefimov S, Yergey AL, Chrambach A*. 2000. *NIH/NICHHD, Macromol Anal Sect, Lab Cellular & Mol Biophys, Bldg 10, Room 9D50, Bethesda, Md 20892, USA. Transfer of SDS-proteins from gel electrophoretic zones into mass spectrometry, using electroelution of the band into buffer without sectioning of the gel. *J Biochem Biophys Methods* **42:** (1-2) 65.

### 12 Protein structural genomics

Alba MM, Sant Ibanez-Koref MF, Hancock JM*. 1999. *Hammersmith Hosp, Imperial Coll, Sch Med, MRC Clin Sci Ctr, Comparat Sequence Anal Grp, Du Cane Rd, London W12 0NN, England. Amino acid reiterations in yeast are overexpressed in particular classes of proteins and show evidence of a slippage-like mutational process. *J Mol Evol* **49:** (6) 789.

Fischer D. 1999. Ben Gurion Univ Negev, Fac Nat Sci, Dept Math &

Copyright © 2000 John Wiley & Sons, Ltd.

Comp Sci, IL-84105 Beer Sheva, Israel. Rational structural genomics: Affirmative action for ORFans and the growth of our structural knowledge. *Protein Eng* **12:** (12) 1029.

Jain R, Ramakumar S*. 1999. *Indian Inst Sci, Dept Phys, IN-560012 Bangalore, India. Stochastic dynamics modeling of the protein sequence length distribution in genomes: Implications for microbial evolution. *Physica A* **273:** (3-4) 476.

Lahr SJ, Broadwater A, Carter CW, Collier ML, Hensley L, Waldner JC, Pielak GJ, Edgell MH*. 1999. *Univ Nth Carolina, Dept Microbiol, CB 7290, Chapel Hill, NC 27599, USA. Patterned library analysis: A method for the quantitative assessment of hypotheses concerning the determinants of protein structure. *Proc Natl Acad Sci U S A* **96:** (26) 14860.

### 13 Metabolomics

Downs DM, Escalante-Semerena JC. 2000. Univ Wisconsin, Dept Bacteriol, Madison, Wi 53706, USA. Impact of genomics and genetics on the elucidation of bacterial metabolism. *Methods* **20:** (1) 47.

### 14 Genomic approaches to development

Cohen-Tannoudji M, Vandormael-Pournin S, Drezen JM, Mercier P, Babinet C, Morello D. 2000. Inst Pasteur, Unite Biol Dev, CNRS, URA 1960, 25 rue Dr Roux, FR-75724 Paris 15, France. *lacZ* sequences prevent regulated expression of housekeeping genes. *Mech Dev* **90:** (1) 29.

Geri C, Turrini A, Giorgetti L, Nicoletti E, Ronchi VN. 1999. CNR, Ist Mutagenesi & Differenziamento, via Svezia 10, Pisa, Italy. Genome plasticity during the acqui™sition of embryogenic competence. *Genome* **42:** (6) 1134.

Kwon JY, Park JM, Gim BS, Han SJ, Lee J*, Kim YJ. 1999. *Yonsei Univ, Dept Biol, 134 Shinchon dong, Seoul 120749, South Korea. *Caenorhabditis elegans* mediator complexes are required for developmental-specific transcriptional activation. *Proc Natl Acad Sci U S A* **96:** (26) 14990.

Luo DL, Oppenheimer DG*. 1999. *Univ Alabama, Dept Biol Sci, Tuscaloosa, Al 35487, USA. Genetic control of trichome branch number in *Arabidopsis*: The roles of the FURCA loci. *Development* **126:** (24) 5547.

Verbeek FJL, Lawson KA, Bard JB. 1999. Netherlands Inst Dev Biol, Hubrecht Lab, Uppsalalaan 8, NL-3584 CT Utrecht, Netherlands. Developmental bioinformatics: Linking genetic data to virtual embryos. *Int J Dev Biol* **43:** (7) 761.

### 15 Technological advances

Atienzar F, Evenden A, Jha A, Savva D, Depledge M. 2000. Univ Plymouth, Dept Biol Sci, Drake Circus, Plymouth PL4 8AA, England. Optimized RAPD analysis generates high-quality genomic DNA profiles at high annealing temperature. *Biotechniques* **28:** (1) 52.

Castro A, Okinaka RT. 1999. Natl Lab, MS-D454, Los Alamos, NM 87545, USA. Ultrasensitive, direct detection of a specific DNA sequence of *Bacillus anthracis* in solution. *Analyst* **125:** (1) 9.

Chen YL, Gan L, Lu ZH. 1999. SE Univ, Natl Lab Mol & Biomol Elect, CN-210096 Nanjing, Peoples Rep China. Using silane coupling reagents in fabrication of DNA arrays on glass support. *Mol Cryst Liq Cryst Sci Tech A* **337:** 477.

Endo Y, Yoshida C, Baba Y*. 1999. *Univ Tokushima, Fac Pharmaceut Sci, Dept Med Chem, Tokushima 770 8505, Japan. DNA sequencing by capillary array electrophoresis with an electric field strength gradient. *J Biochem Biophys Methods* **41:** (2-3) 133.

Fountoulakis M, Takacs MF, Berndt P, Langen H, Takacs B. 1999. Hoffmann La Roche AG, Pharmaceut Res, Genom Technol, Bldg 93-444, CH-4070 Basel, Switzerland. Enrichment of low abundance proteins of *Escherichia coli* by hydroxyapatite chromatography. *Electrophoresis* **20:** (11) 2181.

Garvin AM, Parker KC, Haff L. 2000. Univ Basel, Bioctr, Dept Biochem, CH-4056 Basel, Switzerland. MALDI-TOF based mutation detection using tagged *in vitro* synthesized peptides. *Nat Biotechnol* **18:** (1) 95.

Henrich T, Wittbrodt J. 2000. European Mol Biol Lab, Dev Biol Program, Postfach 102209 Meyerhofstr 1, DE-69012 Heidelberg, Germany. An *in situ* hybridization screen for the rapid isolation of differentially expressed genes. *Dev Genes Evol* **210:** (1) 28.

Keller BO, Li L*. 2000. *Univ Alberta, Dept Chem, Edmonton, Alberta, Canada T6G 2G2. Discerning matrix-cluster peaks in matrix-assisted laser desorption/ionization time-of-flight mass spectra of dilute peptide mixtures. *J Am Soc Mass Spectrom* **11:** (1) 88.

Kwiatkowski M, Fredriksson S, Isaksson A, Nilsson M, Landegren U. 1999. Dept Genet & Pathol, Rudbeck Lab, SE-75185 Uppsala, Sweden. Inversion of *in situ* synthesized oligonucleotides: Improved reagents for hybridization and primer extension in DNA microarrays. *Nucleic Acids Res* **27:** (24) 4710.

Lin H, Hunter JM, Becker CH. 1999. GeneTrace Systems Inc, 1401 Harbor Bay Parkway, Alameda, Ca 94502, USA. Laser desorption of DNA oligomers larger than one kilobase from cooled 4-nitrophenol. *Rapid Commun Mass Spectrom* **13:** (23) 2335.

Liu HJ, Cho BY, Strong R, Krull IS*, Cohen S, Chan KC, Issaq HJ. 1999. *Northeastern Univ, Dept Chem, 102 Hurtig Bldg, 360 Huntington Ave, Boston, Ma 02115, USA. Derivatization of peptides and small proteins for improved identification and detection in capillary zone electrophoresis (CZE). *Anal Chim Acta* **400:** 181.

Puizina J, Javornik B, Bohanec B, Schweizer D, Malusynska J, Papes D. 1999. Univ Split, Fac Nat Sci & Math, Dept Biol, Teslina 12, HR-21000 Split, Croatia. Random amplified polymorphic DNA analysis, genome size, and genomic *in situ* hybridization of triploid viviparous onions. *Genome* **42:** (6) 1208.

Rudi K, Fossheim T, Jakobsen KS. 1999. Matforsk, Norwegian Food Res Inst, Osloveien 1, NO-1430 AS, Norway. Restriction cutting independent method for cloning genomic DNA segments outside the boundaries of known sequences. *Biotechniques* **27:** (6) 1170.

Schutz E, Von Ahsen N. 1999. Univ Hosp, Dept Clin Chem, Robert Kock Str 40, DE-37075 Gottingen, Germany. Spreadsheet software for thermodynamic melting point prediction of oligonucleotide hybridization with and without mismatches. *Biotechniques* **27:** (6) 1218.

Steemers FJ, Ferguson JA, Walt DR*. 2000. *Tufts Univ, Dept Chem, Max Tishler Lab Organ Chem, Medford, Ma 02155, USA. Screening unlabeled DNA targets with randomly ordered fiber-optic gene arrays. *Nat Biotechnol* **18:** (1) 91.

### 16 Bioinformatics

Allison L, Stern L, Edgoose T, Dix TI. 2000. Monash Univ, Sch Comp Sci & Software Engn, Melbourne, Vic 3168, Australia. Sequence complexity for biological sequence analysis. *Comput Chem* **24:** (1) 43.

Baasiri RA, Glasser SR, Steffen DL, Wheeler DA*. 1999. *Baylor Coll Med, Dept Cell Biol, 1 Baylor Plaza, Houston, Tx 77030, USA. The Breast Cancer Gene Database: A collaborative information resource. *Oncogene* **18:** (56) 7958.

Batzoglou S, Berger B, Mesirov J, Lander ES*. 1999. *MIT, Dept Biol, 77 Massachusetts Ave, Cambridge, Ma 02139, USA. Sequencing a genome by walking with clone-end sequences: A mathematical analysis. *Genome Res* **9:** (12) 1163.

Bellgard MI, Hiew HL, Hunter A, Wiebrands M. 1999. Murdoch Univ, Sch Informat Technol, Murdoch Bioinformat Res Grp, Murdoch, WA

Copyright © 2000 John Wiley & Sons, Ltd.

6150, Australia. ORBIT: An integrated environment for user-customized bioinformatics tools. *Bioinformatics* **15:** (10) 847.

Ben Dor A, Shamir R, Yakhini Z. 1999. Univ Washington, Dept Comp Sci & Engn, Seattle, Wa 98105, USA. Clustering gene expression patterns. *J Comput Biol* **6:** (3-4) 281.

Beroud C, Collod-Beroud G, Boileau C, Soussi T, Junien C. 2000. Hop Necker Enfants Malad, INSERM, U383, Clin Maurice Lamy, Pavillon Maurice Lamy, 149-161 rue Sevres, FR-75743 Paris 15, France. UMD (Universal Mutation Database): A Generic software to build and analyze locus-specific databases. *Hum Mutat* **15:** (1) 86.

Brown AF, McKie MA. 2000. MRC, Human Genet Unit, Crewe Rd, Edinburgh EH4 2XU, Scotland. MuStaR™ and other software for locus-specific mutation databases. *Hum Mutat* **15:** (1) 76.

Buldyrev SV, Dokholyan NV, Havlin S, Stanley HE, Stanley RHR. 1999. Boston Univ, Ctr Polymer Studies, Boston, Ma 02215, USA. Expansion of tandem repeats and oligomer clustering in coding and non-coding DNA sequences. *Physica A* **273:** (1-2) 19.

Cotton RGH, Horaitis O. 2000. Mutat Res Ctr, 7th Floor, Daly Wing, 41 Victoria Parade, Fitzroy, Vic 3065, Australia. Quality control in the discovery, reporting, and recording of genomic variation. *Hum Mutat* **15:** (1) 16.

Cuticchia AJ. 2000. Hosp Sick Children, Res Inst, 555 Univ Ave, Toronto, Ontario, Canada M5G 1X8. Future vision of the GDB human genome database. *Hum Mutat* **15:** (1) 62.

Delamarche C, Guerdoux Jamet P, Gras R, Nicolas J. 1999. CNRS UPRES A 6026, Equipe Canaux & Recepteurs Membranaires, Bat 13, Campus Beaulieu, FR-35042 Rennes, France. A symbolic-numeric approach to find patterns in genomes. Application to the translation initiation sites of *E. coli*. *Biochimie* **81:** (11) 1065.

Delcher AL, Harmon D, Kasif S, White O, Salzberg SL. 1999. Loyola Univ, Dept Comp Sci, Baltimore, Md 21210, USA. Improved microbial gene identification with GLIMMER. *Nucleic Acids Res* **27:** (23) 4636.

Fischer S, Crabtree J, Brunk B, Gibson M, Overton GC. 1999. Univ Penn, Ctr Bioinformat, Philadelphia, Pa 19104, USA. bioWidgets: data interaction components for genomics. *Bioinformatics* **15:** (10) 837.

Forst CV, Schulten K. 1999. Univ Illinois, Beckman Inst, Urbana, Il 61801, USA. Evolution of metabolisms: A new method for the comparison of metabolic pathways using genomics information. *J Comput Biol* **6:** (3-4) 343.

Green MK, Johnston MV*, Larsen BS. 1999. *Univ Delaware, Dept Chem & Biochem, Newark, De 19716, USA. Mass accuracy and sequence requirements for protein database searching. *Anal Biochem* **275:** (1) 39.

Klaerr-Blanchard M, Chiapello H, Coward E. 2000. Inst Pasteur, Unite Regulat Express Genet, 28 rue Dr Roux, FR-75724 Paris 15, France. Detecting localized repeats in genomic sequences: A new strategy and its application to *Bacillus subtilis* and *Arabidopsis thaliana* sequences. *Comput Chem* **24:** (1) 57.

Krawczak M, Ball EV, Fenton I, Stenson PD, Abeysinghe S, Thomas N, Cooper DN. 2000. Univ Wales Coll Med, Inst Med Genet, Heath Pk CF14 4XN, Wales. Human gene mutation database: A biomedical information and research resource. *Hum Mutat* **15:** (1) 45.

Lehvaslaiho H, Stupka E, Ashburner M. 2000. European Bioinformat Inst, EMBL Outstation, Wellcome Trust Genome Campus, Cambridge CB10 1SD, England. Sequence variation database project at the European Bioinformatics Institute. *Hum Mutat* **15:** (1) 52.

Ley K, Brewer K, Moton A. 1999. Univ Virginia, Sch Med, Dept Biomed Engn, Health Science Ctr, Box 377, Charlottesville, Va 22908, USA. A Web-based research tool for functional genomics of the microcirculation: The leukocyte adhesion cascade. *Microcirculation* **6:** (4) 259.

Makino S, Amano N, Suzuki M*. 1999. *AIST, NIBHT, CREST Ctr Struct Biol, 1-1 Higashi, Tsukuba, Ibaraki 305 0046, Japan. Visual presentation of complete genomic DNA sequences, and its application to identification of gene-coding regions. *Proc Jpn Acad B* **75:** (10) 311.

Preissner R, Goede A, Frommel C. 1999. Humboldt Univ, Fac Med, Inst Biochem, Monbijoustr 2A, DE-10117 Berlin, Germany. Homonyms and synonyms in the Dictionary of Interfaces in Proteins (DIP). *Bioinformatics* **15:** (10) 832.

Riikonen P, Vihinen M. 1999. Univ Turku, Dept Comp Sci, FI-20520 Turku, Finland. MUTbase: maintenance and analysis of distributed mutation databases. *Bioinformatics* **15:** (10) 852.

Sadegh-Zadeh K. 2000. Univ Munster, Theory Med Dept, Inst Med, Waldeyer St 27, DE-48149 Munster, Germany. Fuzzy genomes. *Artif Intell Med* **18:** (1) 1.

Scordis P, Flower DR, Attwood TK. 1999. Univ Manchester, Sch Biol Sci, Oxford Rd, Manchester M13 9PT, England. FingerPRINTScan: intelligent searching of the PRINTS motif database. *Bioinformatics* **15:** (10) 799.

Scriver CR, Nowacki PM, Lehvaslaiho H. 2000. McGill Univ, Childrens Hosp, Res Inst, Debelle Lab Biochem Genet, 2300 Tupper St, Montreal, Quebec, Canada H3H 1P3. Guidelines and recommendations for content, structure, and deployment of mutation databases: II. Journey in progress. *Hum Mutat* **15:** (1) 13.

Sherry ST, Ward M, Sirotkin K. 2000. NIH/Natl Lib Med, Natl Ctr Biotechnol Informat, Room 8N805, Bldg 38A, Bethesda, Md 20894, USA. Use of molecular variation in the NCBI dbSNP database. *Hum Mutat* **15:** (1) 68.

Stanley HE, Buldyrev SV, Goldberger AL, Havlin S, Peng CK, Simons M. 1999. Boston Univ, Ctr Polymer Studies, Boston, Ma 02215, USA. Scaling features of noncoding DNA. *Physica A* **273:** (1-2) 1.

Tanabe L, Scherf U, Smith LH, Lee JK, Hunter L, Weinstein JN. 1999. 37 Convent Drive, Bldg 37, Rm 5B12, Bethesda, Md 20892, USA. MedMiner: An Internet text-mining tool for biomedical information, with application to gene expression profiling. *Biotechniques* **27:** (6) 1210.

Taylor WR, Saelensminde G, Eidhammer I. 2000. Natl Inst Med Res, Div Math Biol, Mill Hill, Ridgeway, London NW7 1AA, England. Multiple protein sequence alignment using double-dynamic programming. *Comput Chem* **24:** (1) 3.

Thomas GH. 1999. John Innes Ctr, Dept Mol Microbiol, Norwich MR4 7UH, England. Completing the *E. coli* proteome: A database of gene products characterised since the completion of the genome sequence. *Bioinformatics* **15:** (10) 860.

Van Steensel MAM, Celli J, Van Bokhoven JH, Brunner HG*. 1999. *Univ Nijmegen St Radboud Hosp, Dept Human Genet 417, POB 9101, NL-6500 HB Nijmegen, The Netherlands. Probing the Gene eXpression Database for candidate genes. *Eur J Hum Genet* **7:** (8) 910.

Wagner A. 1999. Univ New Mexico, Dept Biol, 167A Castetter Hall, Albuquerque, NM 87131, USA. Genes regulated cooperatively by one or more transcription factors and their identification in whole eukaryotic genomes. *Bioinformatics* **15:** (10) 776.

Wan HH, Wootton JC. 2000. NIH, Computat Biol Branch, Natl Ctr Biotechnol Informat, Bldg 38A, 8th Floor, Bethesda, Md 20894, USA. A global compositional complexity measure for biological sequences: AT-rich and GC-rich genomes encode less complex proteins. *Comput Chem* **24:** (1) 71.

Weissig H, Bourne PE*. 1999. *Univ Calif San Diego, San Diego Supercomp Ctr, 9500 Gilman Dr, La Jolla, Ca 92093, USA. An analysis of the Protein Data Bank in search of temporal and global trends. *Bioinformatics* **15:** (10) 807.

Yuan J, Amend A, Borkowski J, De Marco R, Bailey W, Liu Y, Xie GC, Blevins R. 1999. Merck & Co Inc, Dept Bioinformat, POB 2000, RY80-A1, Rahway, NJ 07065, USA. Multiclustal: A systematic method for surveying Clustal W alignment parameters. *Bioinformatics* **15:** (10) 862.

Copyright © 2000 John Wiley & Sons, Ltd. 
